# Factors Influencing the Outcome of Patients with Primary Ewing Sarcoma of the Sacrum

**DOI:** 10.1155/2024/4751914

**Published:** 2024-03-16

**Authors:** Victor Rechl, Andreas Ranft, Vivek Bhadri, Benedicte Brichard, Stephane Collaud, Sona Cyprova, Hans Eich, Torben Ek, Hans Gelderblom, Jendrik Hardes, Lianne M. Haveman, Wolfgang Hartmann, Peter Hauser, Philip Heesen, Heribert Jürgens, Jukka Kanerva, Thomas Kühne, Anna Raciborska, Jelena Rascon, Arne Streitbürger, Yasmin Uhlenbruch, Beate Timmermann, Josephine Kersting, Minh Thanh Pham, Uta Dirksen

**Affiliations:** ^1^Pediatrics III, West German Cancer Center, University Hospital Essen, University of Duisburg-Essen, Essen, Germany; ^2^German Cancer Consortium, Partnersite, Essen, Germany; ^3^Chris O'Brien Lifehouse, Camperdown, Australia; ^4^Faculty of Medicine and Health, University of Sydney, Camperdown, Australia; ^5^Cliniques Universitaires Saint Luc, Department of Pediatric Hematology and Oncology, Université Catholique de Louvain, Brussels, Belgium; ^6^Lung Clinic, Department of Thoracic Surgery, Cologne Merheim Hospital, University of Witten/Herdecke, Witten, Germany; ^7^Charles University, Motol Child Ren's Hospital, Prague, Czech Republic; ^8^Radiotherapy and Radiooncology, University Hospital Muenster, West German Cancer Center Network, Muenster, Germany; ^9^Childhood Cancer Center Queen Silvia Children's Hospital, Gothenburg, Sweden; ^10^Leiden University Medical Center, Department of Medical Oncology, Leiden, NL, USA; ^11^Clinic of Tumororthopedics, University Hospital Essen, West German Cancer Centre, Essen, Germany; ^12^Princess Máxima Center for Pediatric Oncology, Department of Solid Tumors, Utrecht, Netherlands; ^13^Gerhard Domagk Institute for Pathology, University Hospital Muenster, West German Cancer Center Network, Muenster, Germany; ^14^Velkey László Child's, Health Center, Borsod-Abaúj-Zemplén County University Teaching Hospital, Miskolc, Hungary; ^15^Department of Pediatric Hematology and Oncology, University Children's Hospital Münster, West German Cancer Center Network, Münster, Germany; ^16^HUS Helsinki University Hospital, New Children's Hospital, Div. Hematology and Stem Cell Transplantation, Helsinki, Finland; ^17^Department of Oncology/Hematology, University Children's Hospital Basel, Basel, Switzerland; ^18^Mother and Child Institute, Department of Oncology and Surgical Oncology for Children and Youth, Warsaw, Poland; ^19^Center for Pediatric Oncology and Hematology, Vilnius University Hospital Santaros Klinikos, Vilnius University, Vilnius, Lithuania; ^20^Clinics of Children's Diseases, Faculty of Medicine, Vilnius University, Vilnius, Lithuania; ^21^Patient Representative, St. Josef's Hospital Bochum, University Hospital, Bochum, Germany; ^22^Clinic for Particle Therapy, West German Proton Beam Centre, University Hospital Essen, West German Cancer Centre, German Cancer Research Centre (DKTK), Essen, Germany

## Abstract

**Background:**

Ewing sarcoma (EwS) is a rare and highly malignant bone tumor primarily affecting children, adolescents, and young adults. The pelvis, trunk, and lower extremities are the most common sites, while EwS of the sacrum as a primary site is very rare, and only few studies focusing on this location are published. Due to the anatomical condition, local treatment is challenging in sacral malignancies. We analyzed factors that might influence the outcome of patients suffering from sacral EwS.

**Methods:**

We retrospectively analyzed data of the GPOH EURO-E.W.I.N.G 99 trial and the EWING 2008 trial, with a cohort of 124 patients with localized or metastatic sacral EwS. The study endpoints were overall survival (OS) and event-free survival (EFS). OS and EFS were calculated using the Kaplan–Meier method, and univariate comparisons were estimated using the log-rank test. Hazard ratios (HRs) with respective 95% confidence intervals (CIs) were estimated in a multivariable Cox regression model.

**Results:**

The presence of metastases (3y-EFS: 0.33 vs. 0.68; *P* < 0.001; HR = 3.4, 95% CI 1.7 to 6.6; 3y-OS: 0.48 vs. 0.85; *P* < 0.001; HR = 4.23, 95% CI 1.8 to 9.7), large tumor volume (≥200 ml) (3y-EFS: 0.36 vs. 0.69; *P*=0.02; HR = 2.1, 95% CI 1.1 to 4.0; 3y-OS: 0.42 vs. 0.73; *P*=0.04; HR = 2.1, 95% CI 1.03 to 4.5), and age ≥18 years (3y-EFS: 0.41 vs. 0.60; *P*=0.02; HR = 2.6, 95% CI 1.3 to 5.2; 3y-OS: 0.294 vs. 0.59; *P*=0.01; HR = 2.92, 95% CI 1.29 to 6.6) were revealed as adverse prognostic factors.

**Conclusion:**

Young age seems to positively influence patients` survival, especially in patients with primary metastatic disease. In this context, our results support other studies, stating that older age has a negative impact on survival. Tumor volume, metastases, and the type of local therapy modality have an impact on the outcome of sacral EwS. Level of evidence: Level 2. This trial is registered with NCT00020566 and NCT00987636.

## 1. Introduction

Ewing sarcoma (EwS) is a rare and highly malignant bone and soft tissue tumor that mainly affects children, adolescents, and young adults with a median age of 15 years at the time of diagnosis [1–4]. Histologically, EwS presents as nests of small round blue cells and is molecularly characterized by EwSR1 gene rearrangements [3, 5–7]. The general annual incidence is about one to three per million population with a slight predominance in males and a significantly higher occurrence in Caucasians than in people of color [5, 8–10]. Metastases are detected in 25% of the patients at the time of diagnosis and predominately manifest in the lungs, followed by bone or rather bone marrow [11–13]. Among patients with metastatic disease, pulmonary metastases are associated with a better outcome than extrapulmonary [1, 13–18]. In the past decades, the outcome in localized EwS has been improved due to the implementation of a multimodal therapeutic approach with systemic chemotherapy in combination with local therapy (LT), while disseminated disease and relapse are linked to low survival rates [1, 14, 16, 19–22]. In most sites, surgery as LT modality is associated with a favorable outcome, whenever possible. However, complete resection with wide margins is not achievable at all sites, and optimal local treatment for difficult sites such as the sacrum is viewed to exhibit the biggest benefit for patients, but the anatomical location is decisive, and due to the proximity to nerve roots and vascular structures, surgery of the sacrum is a very challenging entity [14, 23–32]. Already well-known influencing factors for EwS are sex, age, metastasis, tumor volume, local treatment modality, anatomic site of the primary tumor, resection margins, histological response, and relapse [18, 28, 31, 33]. Besides the individual patient-associated factors, it is crucial to mention that there are also tumor-related factors on a cellular and genetic level such as the tumor's microenvironment and genetic rearrangements, which determine its ability to escape the immune system and have an impact on the metastasis behavior [34, 35]. There are many scientific papers dealing with EwS affecting the extremities or the innominate bones, but scientific work on EwS of the sacrum and the outcome of known prognostic factors of this specific localization is only scarcely available. Most publications subsume sacral EwS under pelvic EwS, and therefore, we focused our analysis on the sacrum only and analyzed if there are certain factors that have an impact on survival for this patient group. Confirmed as negative prognostic factors were metastases at the time of diagnosis (3y-EFS: 0.33 vs. 0.68; *P* < 0.001; HR = 3.4, 95% CI 1.7 to 6.6; 3y-OS: 0.48 vs. 0.85; *P* < 0.001; HR = 4.23, 95% CI 1.8 to 9.7), large tumor volume (≥200 ml) (3y-EFS: 0.36 vs. 0.69; *P*=0.02; HR = 2.1, 95% CI 1.1 to 4.0; 3y-OS: 0.42 vs. 0.73; *P*=0.04; HR = 2.1, 95% CI 1.03 to 4.5), and age ≥18 years (3y-EFS: 0.41 vs. 0.60; *P*=0.02; HR = 2.6, 95% CI 1.3 to 5.2; 3y-OS: 0.294 vs. 0.59; *P*=0.01; HR = 2.92, 95% CI 1.29 to 6.6). Patients with infestation of the lower parts of the sacrum had a better outcome than patients with higher affected sacral levels (3y-EFS: 0.74 vs. 0.31; *P*=0.54; 3y-OS: 0.89 vs. 0.38; *P*=0.006).

## 2. Methods

### 2.1. The Patient Collective

The analysis included patients that presented with an untreated primary EwS of the sacrum. Patients who presented with an EwS of neighboring structures (i.e., iliac crest) infiltrating the sacrum were excluded. A total of 124 patients were included in the analysis, comprising localized disease (*N* = 70) and metastatic disease (*N* = 53). In one patient, the stage of disease was unknown. Data from two international clinical trials (Society for Pediatric Oncology and Hematology (GPOH) EURO E.W.I.N.G-99 (NCT00020566) and Ewing 2008 (NCT00987636)) were screened. EwS was confirmed by pathology and molecular diagnostics in all patients. Informed consent was obtained from all patients. The details of the treatment procedures are described precisely in the corresponding treatment protocols (NCT00020566 and NCT00987636) [15, 36–41].

### 2.2. Treatment Schemes

Patients in both trials received six courses of vincristine, ifosfamide, doxorubicin, and etoposide (VIDE) as induction chemotherapy [42]. In both protocols, patients were stratified to a randomized treatment arm with an affiliated consolidation chemotherapy according to risk groups defined by staging criteria and tumor characteristics such as histological response to induction chemotherapy and tumor volume. Consolidation treatment of the R2-arms in both trials was the same, while R1 in EWING 2008 received either VAC (vincristine, actinomycin D, and cyclophosphamide) for female patients or VAI (vincristine, actinomycin D, and ifosfamide) for male patients and additional randomization concerning administration of zoledronic acid. While the trial from 1999 divided the consolidation therapy into Treo-Mel (treosulfan + melphalan), Bu-Mel (busulfan + melphalan), or phase 2, the trial from 2008 compared the group VAC-only with Vac + Treo-Mel. In the entire cohort and all sites, surgery as a local treatment was performed whenever feasible, aiming for complete surgical removal of the tumor with wide margins. In case of insufficient margins or poor histological response (≥10% viable tumor cells in the specimen), radiotherapy was recommended in addition to surgery [43]. The Ewing 2008 trial protocol furthermore recommended postoperative radiotherapy for patients with large pelvic tumors even if wide resection margins were obtained [25]. Preoperative radiotherapy was performed, if progression of the tumor was seen or if wide resection margins were not possible to obtain. Definitive radiotherapy was indicated, if the tumor was inoperable or if it was located at anatomical sites, where surgery would lead to severe complications or would lead to mutilation of the patient. The recommended preoperative radiotherapy dose was 54.4 Gy and the definitive dose was 54 Gy for the Euro E.W.I.N.G 99 protocol, while the preoperative dose was 54 Gy, and the definitive dose was 54.4 Gy for the Ewing 2008 trial. Both the protocols administered 45–54 Gy depending on histological response and the obtained surgical margins as a postoperative radiation dose, if necessary.

### 2.3. Data Collection and Statistical Analysis

Data on patient demographics, tumor characteristics, local treatment modality, adjuvant treatment modality, follow-up, status, and applied study protocol were collected, coded, and entered into an electronic database. Endpoints of this retrospective analysis were event-free survival (EFS) and overall survival (OS).

We combined the patients and harmonized data from both trials into one collective and analyzed the dataset. Statistical analyses were carried out with IBM SPSS statistics for Macintosh, Version 28.0. Armonk, NY: IBM Corp (IBM Corp. Released 2021). Overall survival (OS) and event-free survival (EFS) were calculated using the Kaplan–Meier method. OS time was defined as the interval between the date of diagnostic biopsy and date of death or last follow-up. Time for EFS was defined as an interval between the date of diagnostic biopsy and the date of a first event. An event was defined as death, progression of disease, relapse, secondary malignancy, or the occurrence of new metastases. Survivors were censored at the date of last contact. Univariate comparisons were estimated using the log-rank test. We tested the proportional hazards assumption before performing the Cox regression. The survival analysis was based on follow-up data with cutoff July 2021, and hazard ratios (HRs) with respective 95% confidence intervals (CIs) were estimated in a multivariable Cox regression model. Well-known factors for EwS associated with survival, e.g., age, sex, tumor volume, local treatment modality, and applied study protocol were included in the univariable and multivariable analyses [18, 28, 31]. Chi-square or Fisher's test was used for examining the proportions. No alpha corrections were performed for multiple testing because of the exploratory nature of the analysis. The *P* value was relevant if *P* < 0.05 (two sided).

## 3. Results

Between 1998 and 2009, 1471 patients with untreated histologically confirmed EwS were registered in the GPOH database of the EURO E.W.I.N.G-99 trial [25]. Of the 1471 patients, 4.7% (69 of 1471) presented with a primary sacral EwS, and of them, 39% (27 of 69) presented with metastases at diagnosis and 61% (42 of 69) with localized disease. The Ewing 2008 trial registered 1421 patients between 2009 and 2019, and 3.9% (55 of 1421) presented with a primary sacral EwS; of them, 49% (27 of 55) presented with metastases at diagnosis and 51% (28 of 55) with localized disease. Patients with missing data were excluded from the analyses of the respective variables. The median range of follow-up for the whole patient collective was 39 months (1 to 217) and 66 months (13 to 217) for patients that were still alive at the time of the last follow-up.

**Figure 1:**
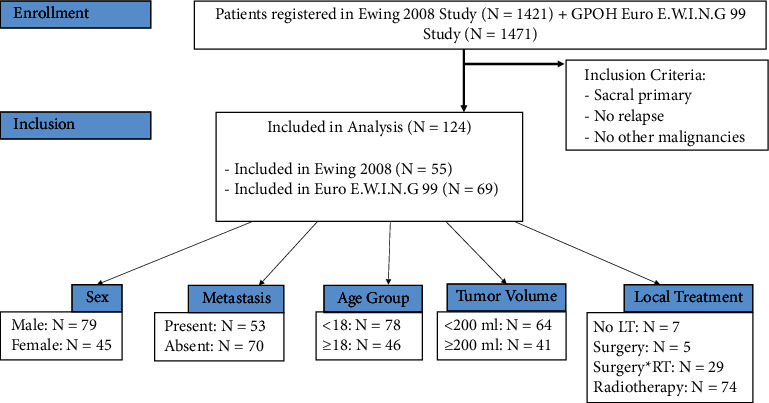
Flowchart showing the selection process of our patient collective and the descriptive variables we analyzed with the corresponding *N* of each variable.

A brief description of the selection process can be seen in Figure 1 and general patient demographics in Table 1. The study population consisted of 63.7% (79 of 124) male and 36.3% (45 of 124) female patients. The median age at diagnosis was 17 years (range = 0.02 to 71.99). By splitting the patients into age groups, we obtained one which was defined to be <18 years and the other ≥18 years. An estimated tumor volume categorization was available in 105 patients, which ranged from 1 mL to 2097 mL. Large tumor volume was defined as tumors ≥200 ml. Data on the precise sacral levels affected were documented in 38.7% of the cases (48 of 124), and for a better comparison, we divided them into two groups. The first group includes patients with the affected sacral levels S1 and above, while the second group includes any level below S1. No local therapy was carried out in 5.6% (7 of 124) of the patients, mainly due to early progression. No data on the local therapy modality were available in 7.3% (9 of 124) of the patients. Surgical margins were radical in 40% (14 of 35), marginal in 20% (7 of 35), and intralesional in 40% (14 of 35). Regarding radiation therapy, 42.7% (52 of 124) received high voltage X-ray radiation, 12.9% (16 of 124) protons, and 0.8% (1 of 124) cobalt-60.

**Figure 2:**
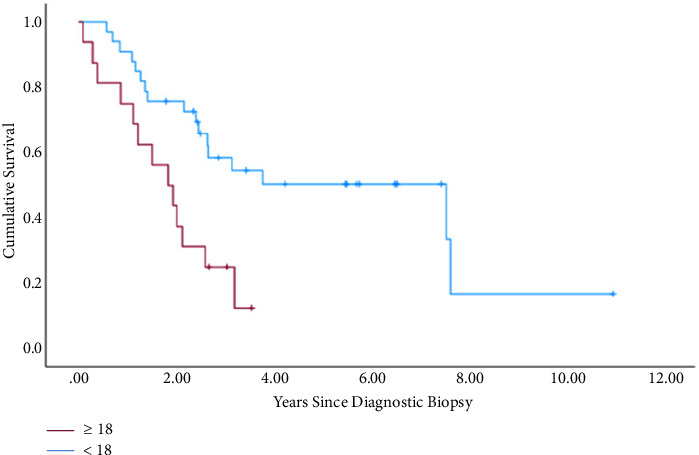
OS for different age groups (<18 years: *N* = 33; ≥18 years: *N* = 17) within the metastasized subgroup, *P*=0.01.

**Figure 3:**
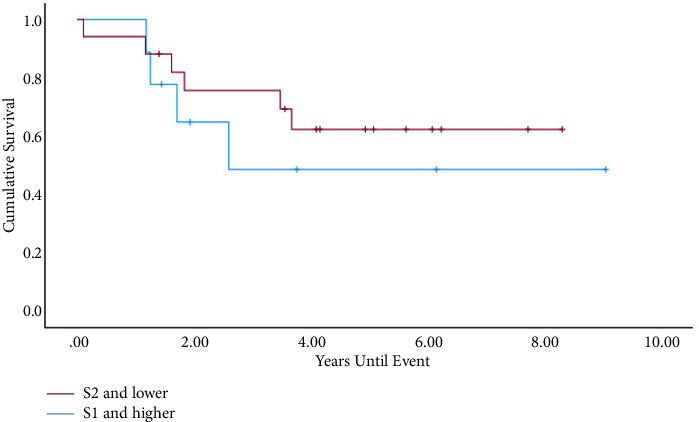
EFS for different affected sacral levels within the localized subgroup (S1 and higher: *N* = 9; S2 and lower: *N* = 19), *P*=0.041.

**Figure 4:**
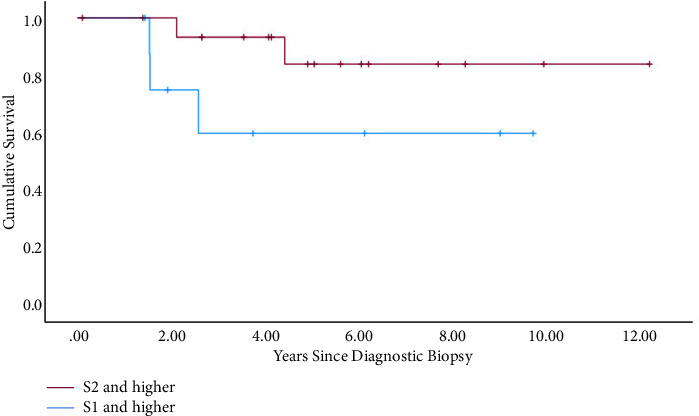
OS for different affected sacral levels within the localized subgroup (S1 and higher: *N* = 9; S2 and lower: *N* = 19), *P*=0.001.

3y-EFS for the whole patient collective (*N* = 24) was 0.53, and 3y-OS was 0.69. The outcome was better in the group <18 years (*N* = 78) (3y-EFS: 0.6 vs. 0.41; *P* = 0.10; 3y-OS: 0.76 vs. 0.59; *P* = 0.043) (Figure 2). Within the metastasized subgroup (*N* = 53), pulmonary manifestation (*N* = 35) in contrast to extrapulmonary (*N* = 17) also showed better rates (3y-EFS: 0.43 vs. 0.12; *P* = 0.023; 3y-OS: 0.58 vs. 0.29; *P* = 0.04). The data of patients with documented affected sacral levels showed that the outcome was remarkably better in patients with infestation of the lower parts of the sacrum (*N* = 32) (3y-EFS: 0.74 vs. 0.31; *P* = 0.54; 3y-OS: 0.89 vs. 0.38; *P* = 0.006) (Figure 3 and 4). Tumor volume ≥200 ml (*N* = 41) in contrast to <200 ml (*N* = 64) was associated with a worse outcome (3y-EFS: 0.36 vs. 0.68; *P* = 0.002; 3y-OS: 0.59 vs. 0.82; *P* = 0.001). For local therapy modality, the best results were achieved with the combined surgery + radiotherapy (*N* = 29) (3y-EFS: 0.71; 3y-OS: 0.82).

**Figure 5:**
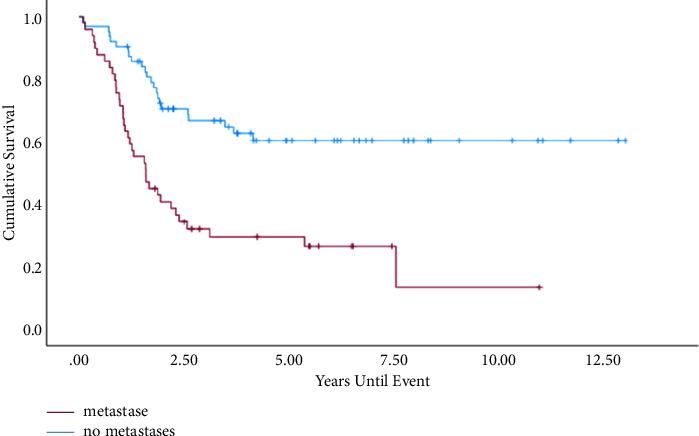
EFS for patients with (*N* = 50) and without (*N* = 65) metastases at the time of diagnosis, *P* < 0.001.

Regarding event-free survival, the 3y-EFS for localized disease is 0.68 and 0.33 for patients with metastases at diagnosis (Figure 5). Tumor volume ≥200 ml was associated with a significantly lower EFS and OS in both localized and systemic disease (3y-EFS; 0.79 vs. 0.25; *P*=0.02; 3y-OS; 0.53 vs. 0.48; *P* < 0.001). A major difference regarding the outcome was observed in patients with definitive radiotherapy compared to other patients. This was apparent by a higher EFS in localized disease in contrast to patients with disseminated disease (3y-EFS: 0.75 vs. 0.3; *P* < 0.001).

By performing multivariable analyses, the presence of metastases (3y-EFS: 0.33 vs. 0.68; *P* < 0.001; HR = 3.4, 95% CI 1.7 to 6.6; 3y-OS: 0.48 vs. 0.85; *P* < 0.001; HR = 4.23, 95% CI 1.8 to 9.7), large tumor volume (3y-EFS: 0.36 vs. 0.69; *P*=0.02; HR = 2.1, 95% CI 1.1 to 4.0; 3y-OS: 0.42 vs. 0.73; *P*=0.04; HR = 2.1, 95% CI 1.03 to 4.5), and age ≥18 years (3y-EFS: 0.41 vs. 0.60; *P*=0.02; HR = 2.6, 95% CI 1.3 to 5.2; 3y-OS: 0.294 vs. 0.59; *P*=0.01; HR = 2.92, 95% CI 1.29 to 6.6) were revealed as adverse prognostic factors (Table 2).

## 4. Discussion

The pelvis itself represents one of the most common sites for EwS, while the sacrum as a primary site is rare [5, 18, 23, 44, 45]. In comparison to the pelvis, sacral manifestation is associated with a better prognosis, and one reason may be the smaller tumor volume at the time of diagnosis [2, 5, 18, 19, 23–25]. Another important finding in our analysis was that patients with affected sacral levels of S2 or lower exhibit a much better OS than S1 and higher (3y-OS: 0.89 vs. 0.38; *P*=0.01). The patients with lower affected sacral levels received combined therapy more often (*N* = 12) than with higher affected sacral levels (*N* = 3). So, apparently, not only the sacrum itself poses a better outcome in contrast to the pelvis [23, 25], but also the affected anatomical level within the sacrum has an impact on survival. Due to the anatomic site, planning of local treatment is challenging. Our retrospective analysis was performed on patients that were prospectively included into international clinical trials. Both trials were built on a similar chemotherapy backbone. Thus, confounding effects of major variations in therapeutic concepts are minimized. Our findings regarding local therapy modality for the localized patient collective support the results of previous papers by Andreou et al. [25] or by Hesla et al. [23] with the Scandinavian sarcoma group. Hesla et al. analyzed 117 patients in total, of which 29 patients suffered from sacral EwS and 88 patients had EwS of innominate bones. They state that sacral EwS has a better outcome than EwS of the innominate bones and that radiotherapy seems to be the most suitable therapy modality for sacral EwS. The study performed by Andreou et al. analyzed 180 patients with localized pelvic EwS, of which 40 patients had a sacral primary. The results also showed an improved outcome and local control for sacral primaries of EwS. The outcome for radiotherapy alone was equal to the combined therapy. On the other hand, a study performed by Jawad et al. [46] with 135 nonmetastatic patients of the Surveillance, Epidemiology, and End Result (SEER) database and 185 nonmetastatic patients of the National Cancer Database (NCDB) showed the best results for localized disease if only surgery was applied as local treatment modality [46]. The latter did however not discriminate between sacral and pelvic tumors. No detailed analysis of sacral tumors was provided, and the difference in patient characteristics may have led to a different outcome. The question whether to use a combined therapy or definitive radiotherapy in localized and metastasized patients with sacral primary is still not unambiguous due to this rare manifestation. A congruent statement of how to deal with this entity might soon be possible by the help of the HIBISCus project of which we are a part of as CESS [47]. For patients with primary metastatic disease, our results indicate that a combined treatment approach is the treatment with the best outcome in terms of survival, provided that a surgical intervention is feasible. The presence of metastases is well known to be the main factor for a dismal outcome in patients with EwS which was also confirmed in our collective [2, 14, 28, 38]. In our cohort, the majority of metastasized patients (*N* = 35) had pulmonary lesions only which are known to have a better outcome than other metastatic sites, i.e., bone [13, 15]. It is important to keep that in mind while comparing the outcome of our metastatic patients with the patients' outcome of other studies with similar cohorts, as a possible reason for better survival. Furthermore, we compare our cohort with known prognostic factors. Young age, in our case <18 years, showed a more favorable outcome regardless of disease stage. We set this age limit as it has been described that patients suffering from EwS of the pelvic area are older than patients with other affected localizations, a finding that is also confirmed in by our study [48]. Young age in general is associated with a better outcome, and the reason for this may be influenced by factors such as physiology, tumor biology, and response to treatment [18, 20]. Early tumor detection could contribute to smaller tumor volumes, which would lead to better survival rates [3, 29, 40]. As described for other sites, our cohort also showed a better outcome in patients with small tumor volume <200 ml in both the localized and metastatic groups. Whether gender has an impact on the outcome of patients with EwS is still controversially discussed. While some studies show improved survival rates for female patients [12, 49, 50], others state that there is no impact on survival at all [51]. Our findings indicate that there is no difference regarding EFS or OS between female and male patients with a sacral primary. The positive influence of female gender on survival in some studies might be explained by a disproportion within the age groups as described in the study of Cotterill et al. Even though we were able to analyze local treatment modalities and prognostic factors in a substantial number of patients, numbers remain small, and we believe that international collaboration is mandatory to pave the way for a better outcome in EwS.

## 5. Conclusion

This article provides a substantial number of patients suffering from primary EwS of the sacrum and tries to help physicians to categorize the possible outcome of this patient group. The rare occurrence of sacral EwS leads to a scarce data situation in research about this topic, and most of the available publications about sacral EwS analyze small numbers of patients or subsume it under pelvic EwS including the innominate bones. Our analysis addresses the sacrum as primary only, to find out about how certain factors influence the outcome of affected patients. Young age, tumor volume <200 ml, localized disease stadium, and affected sacral levels of S2 and lower are factors associated with the most favorable outcome regarding OS and EFS. The patients' sex seemed to have no influence on the outcome. Regarding local therapy modality, the anatomical location is decisive, and local therapy must be chosen individually.

## Figures and Tables

**Table 1:** Descriptive statistics of the patient collective.

Patient characteristics		*N* (total *N* = 124)	%
Age	<18	78	62.9
	≥18	46	37.1

Gender	Male	79	63.7
	Female	45	36.3

Tumor volume	<200 mL	64	51.6
	≥200 mL	41	33.1
	Unknown	19	15.3

Metastases at diagnosis	Yes	53	42.7
	No	70	
	Unknown	1	
	Pulmonary	35	57.3
	Extrapulmonary	17	

Local therapy	No local therapy	7	5.6
	Surgery only	5	4
	Radiotherapy only	74	59.7
	Surgery + radiotherapy	29	23.4
	Unknown	9	7.3

Study protocol	GPOH EE99	69	55.6
	EWING 2008	55	44.4

Affected sacral levels	S1 and higher	16	12.9
	S2 and lower	32	25.8
	Unknown	76	61.3

**Table 2:** Multivariate analysis with the Cox proportional hazards model of overall survival in patients with primary EwS of the sacrum (main analysis was OS).

	*P*	Hazard ratio	95% confidence interval	
			Lower	Upper
Sex (male/female)	0.26	1.58	0.71	3.52
Metastases at diagnosis	<0.001	4.23	1.84	9.70
Tumor volume (≥200 mL/<200 mL)	0.04	2.14	1.03	4.46
Study protocol (GPOH EE99/EWING2008)	0.25	1.64	0.70	
Age group (≥18 years/<18 years)	0.01	2.92	1.29	6.60
Surgery	0.06			
Surgery *∗* radiotherapy	0.02	0.17	0.04	0.74
Radiotherapy	0.10	0.33	0.09	1.21

## Data Availability

The clinical database-derived data used to support the findings of this study are included in the article.
